# The landscape of genetic variations in non-syndromic primary ovarian insufficiency in the MENA region: a systematic review

**DOI:** 10.3389/fendo.2023.1289333

**Published:** 2024-04-26

**Authors:** Asma Allouch, Tara Al-Barazenji, Mashael Al-Shafai, Atiyeh M. Abdallah

**Affiliations:** ^1^ Department of Biomedical Sciences, College of Health Sciences, QU Health, Qatar University, Doha, Qatar; ^2^ Biomedical Research Center, Qatar University, Doha, Qatar

**Keywords:** primary ovarian insufficiency, Arab, MENA, genetic variant, systematic review

## Abstract

**Introduction:**

Premature ovarian insufficiency (POI) is a primary cause of infertility with variable clinical manifestations. POI is a multifactorial disease with both environmental and known genetic etiologies, but data on the genetic variations associated with POI in the Middle East and North Africa (MENA) region are scarce. The aim of this study was to systematically review all known genetic causes of POI in the MENA region.

**Methods:**

The PubMed, Science Direct, ProQuest, and Embase databases were searched from inception to December 2022 for all reports of genetic variants associated with POI in the MENA region. Clinical and genetic data were collected from eligible articles, and ClinVar and PubMed (dbSNP) were searched for variants.

**Results:**

Of 1,803 studies, 25 met the inclusion criteria. Fifteen studies were case-control studies and ten were case reports representing 1,080 non-syndromic POI patients in total. Seventy-nine variants in 25 genes associated with POI were reported in ten MENA countries. Of the 79 variants, 46 were rare and 33 were common variants. Of the 46 rare variants, 19 were pathogenic or likely pathogenic according to ACMG classification guidelines and ClinVar. No clear phenotype-genotype association was observed. Male family members carrying pathogenic variants also had infertility problems.

**Discussion:**

To our best knowledge, this is the first systematic review of the genetic variants associated with POI in the MENA region. Further functional studies are needed to assess the disease-causing molecular mechanisms of these variants. Knowledge of the genetic basis of POI in the Middle East could facilitate early detection of the condition and thus early implementation of therapeutic interventions, paving the way for precision medicine options in specific populations.

## 1 Introduction

Primary ovarian insufficiency (POI) is a complex and significant medical condition characterized by the abrupt and permanent cessation of menstrual periods in women under the age of 40(PMID 36630623). This condition leads to a unique physiological state, characterized by hypergonadotropic hypoestrogenic amenorrhea, which in turn is a consequence of the depletion of the ovarian follicular pool. POI can manifest itself either before puberty, referred to as primary amenorrhea, or after the onset of menstrual cycles, termed secondary amenorrhea. (PMID 32934798).

Studying POI is of paramount importance as it has far-reaching implications for the affected individuals’ reproductive health, general well-being, and long-term health outcomes. Not only does it signify the loss of fertility, but it also raises concerns about hormonal imbalances, bone health, and cardiovascular risks. Additionally, POI may be a symptom of underlying genetic, autoimmune, or environmental factors that can impact a woman’s overall health. A deeper understanding of POI is essential for early diagnosis, targeted treatment, and support to enhance the quality of life for those affected. This introduction sets the stage for a more comprehensive exploration of the multifaceted aspects of POI, emphasizing its importance in both clinical and research contexts. (PMID33427510).

According to the European Society of Human Reproduction and Embryology (ESHRE), the diagnostic criteria for POI are age, cycle irregularities for four months or more (oligo/amenorrhea), and elevated follicle-stimulating hormone (FSH) levels (>25 IU/L) (1). POI affects around 1% of women aged between 35 and 40 years and 0.1% of women younger than 30 years worldwide. Fifteen percent of patients report a family history of POI, underscoring the strong genetic etiology of the condition (2). Indeed, POI is multifactorial and clinically heterogeneous, and most POI cases are idiopathic or spontaneous, with genetic, autoimmune, and environmental factors contributing to its pathoetiology. Additionally, it has been shown that syndromic POI accounts for 10-20% of POI cases: Turner syndrome in 4-5% of cases and Fragile X syndrome (*FMR1* permutations) in 3-15% (3). Twin studies have estimated that the heritability of POI is 53-71%, and up to 40% of POI cases are reported to have genetic causes. This systematic review focuses on reported non-syndromic POI cases, where the majority of variants identified in non-syndromic POI from consanguineous families have been detected in genes important for meiosis, homologous recombination, as well as DNA damage and repair (4).

Relatively recent next-generation (NGS) and whole-exome sequencing (WES) efforts of large POI families have identified new genetic variants associated with the disease (5). However, few have been functionally validated as causative (6). According to the Online Mendelian Inheritance in Man (OMIM) database, *BNC1, NOBOX*, and *NR5A1* show autosomal dominant inheritance; *FANCM, GDF9, HFM1, HSF2BP, MCM9, MRAPS22, MSH4, MSH5, STAG3, SPIDR, SYCE1*, and *ZSWIM7* show autosomal recessive inheritance; and *BMP15* shows X-linked inheritance in non-syndromic POI. However, POI genetics is believed to be much more complex than participation of these genes alone (7).

To our best knowledge, this is the first systematic review of the genetic variations associated with non-syndromic POI in the MENA region. Knowledge about the genetic basis on POI in the Middle East paves the way for implementation of precision medicine strategies in this population.

## 2 Methods

### 2.1 Search strategy and objectives

This search strategy for this review followed the Preferred Reporting Items for Systematic Review and Meta-Analysis (PRISMA) guidelines (8). The primary objective of this study was to determine the genetic variations associated with POI in populations across the MENA region. There are several MENA region definitions but, in this review, we followed the Michael and Staley definition, which includes the Arab countries (Qatar, Saudi Arabia, Somalia, Sudan, Syria, Tunisia, United Arab Emirates, Algeria, Bahrain, Djibouti, Egypt, Iraq, Jordan, Kuwait, Lebanon, Libya, Mauritania, Morocco, Oman, Palestine and Yemen) in addition to Turkey and Iran (9). The secondary objective was to report the variability in clinical phenotypes observed in patients diagnosed with POI.

The PubMed, Science Direct, ProQuest, and Scopus databases were searched from inception to October 2022 using key phrases constructed to include both primary and secondary outcomes. The keywords included the terms “primary ovarian insufficiency” OR “premature ovarian failure” AND “North African and Middle Eastern countries” AND “mutation”, “gene”, “variant”, or “polymorphism”. The abstracts and titles of all retrieved articles were initially screened, and articles satisfying the inclusion criteria were then thoroughly evaluated and included in the final analysis.

### 2.2 Study selection

**Figure 1:**
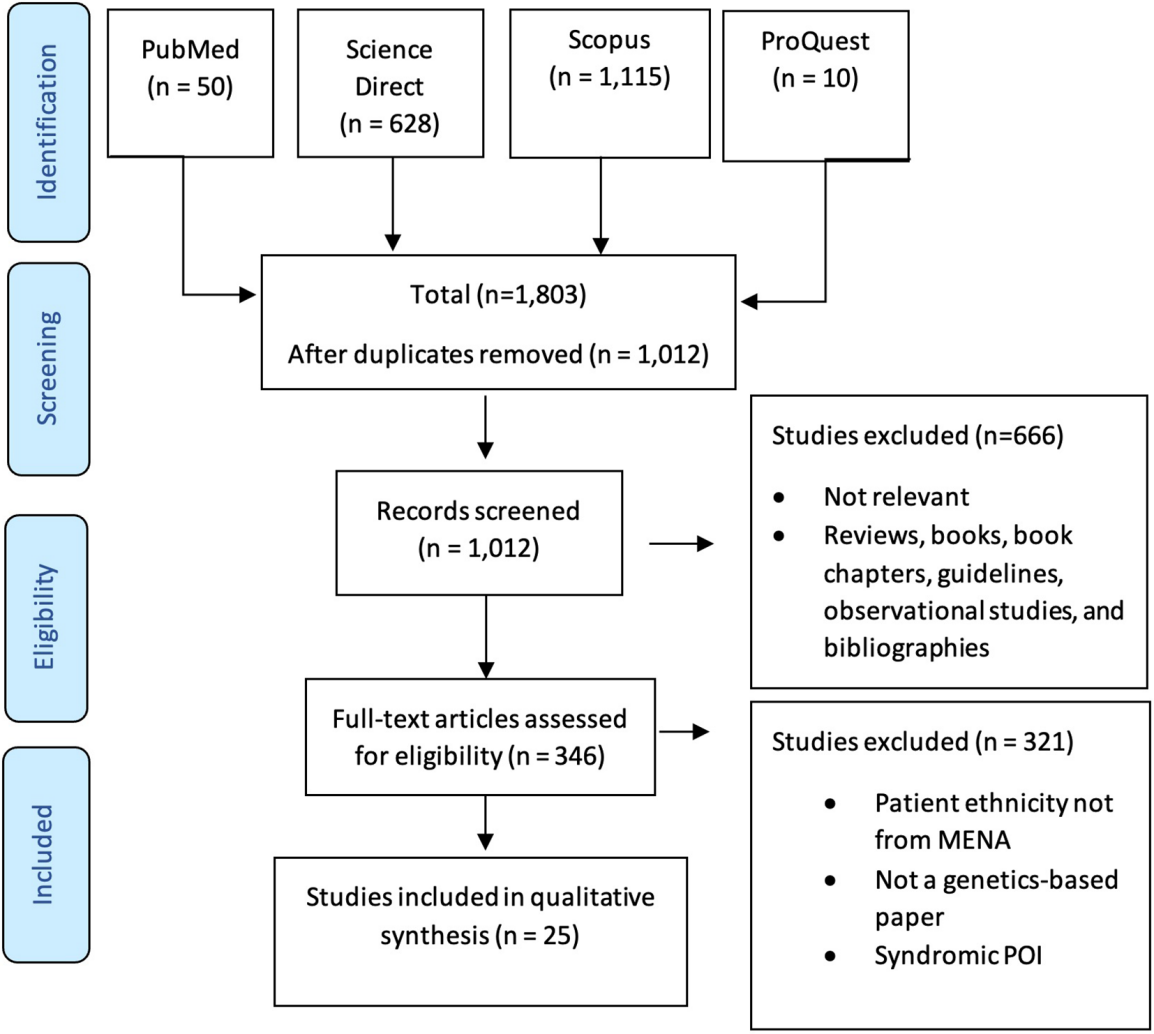
The screening and selection process used in this study according to PRISMA guidelines.

Two researchers evaluated the retrieved records, and discrepancies was resolved through a consensus with the senior author. The total number of hits from each database was documented. Research articles meeting the following inclusion criteria according to the PICOS statement (**Table 1**) were fully assessed: 1) publication in a peer-reviewed journal; 2) population from a MENA country according to the Michael and Staley definition (9); and 3) the article explored genetic variants associated with POI. Any research papers that lacked genetic variants associated with POI, studied a population not from a MENA country, or that were reviews, books, protocols, or guidelines were excluded (**Table 1**). After eliminating duplicates, titles and abstracts of the remaining articles were reviewed, and any record not matching the inclusion criteria was excluded. The full text of these eligible papers was assessed to collect relevant records. All assessments were performed by two researchers with the assistance of the senior author to resolve any disagreements. The screening and selection process in shown in **Figure 1**.

**Table 1:** Inclusion and exclusion criteria according to the PICOS statement*.

	Included	Excluded
**Population**	MENA region	Other populations
**Interventions**	None	None
**Comparators**	POI patients with genetic variation	Articles with no genetic analysis
**Study design**	Prospective, retrospective cohort, case reports, and research articles	Reviews, books, protocols, guidelines, and animal studies
**Primary outcome**	Genetic variants reported in POI patients	Irrelevant
**Secondary outcomes**	Clinical phenotype variability in POI patients	Irrelevant

PICO, Population, Intervention, Comparator, Outcomes, and Study design, POI: primary ovarian insufficiency.

### 2.3 Quality control assessment and data extraction

Risk of bias in eligible articles was assessed by two researchers using the Quality Assessment of Diagnostic Accuracy Studies-2 (QUADAS-2) tool (10), which rates sections as “low risk”, “high risk”, or “unclear risk”. Discrepancies were resolved with the senior author opinion. Data items were extracted from both tables and text of eligible articles and compiled in a Microsoft Excel spreadsheet. Two researchers reviewed data accuracy. Gene name, variant, sample size, genetic test used, number of patients tested, proportion with the causative variant, zygosity, consanguinity, gender, and phenotypic information were extracted. (PMID25741868).

### 2.4 Analysis of retrieved genetic variants

Several databases have been consulted to further investigate all the retrieved genetic variants from the 25 included studies. Namely, NCBI ClinVar (https://www.ncbi.nlm.nih.gov/clinvar/), NCBI (dbSNP) (https://www.ncbi.nlm.nih.gov/snp/), and Genome Aggregation Database (gnomAD) to obtain the minor allele frequency (MAF) (https://gnomad.broadinstitute.org/). Based on the minor allele frequency in gnomAD, retrieved variants were categorized into rare and common variations, where rare variants were found in less than or equal to 1% of gnomAD population (MAF ≤ 0.01), and common variants were defined as having allele frequency higher than 1% (MAF > 0.01) (PMID: 35117776). Further, to interpret the retrieved variants, the standards and guidelines for the clinical Interpretation of genetic variants by the American College of Medical Genetics and the Association for Molecular Pathology (ACMG/AMP) of 2015 were followed (via https://wintervar.wglab.org/). The ACMG/AMP guidelines entail classifying genetic variants into five categories by integrating several of the typical forms of variant evidence (e.g., population data, computational data, functional data, segregation data), these categories are: Pathogenic (P), Likely Pathogenic (LP), Variant of Uncertain Significance (VUS), Likely Benign (LB), or Benign (B).(PMID: 25741868).

## 3 Results

### 3.1 Search outcome

Our search yielded 1,803 studies, of which 1,012 remained after removal of duplicates. The remaining articles were subjected to primary screening by title, abstract, and PICOS assessment for eligibility according to the inclusion and exclusion criteria. Of the screened articles, 346 were eligible for full assessment. Of these, 315 articles were either not genetic studies, patient ethnicity was not from the MENA region, or the study included syndromic POI. Therefore, 25 articles were eligible for inclusion in this review. **Table 2** summarizes the included articles.

**Table 2:** Description of the included articles with genetic variants reported in the MENA populations.

No	Reference	Population	Study type	Detection Method	Sample description	Reported genes
1	Afkhami et al., 2022 (11)	Iran	Case - control	TGS	24 patients	*BMP15*
2	Akbari et al., 2022 (12)		Case report	WES	1 family, 3 patients	*STAG3*
3	Mehrjooy et al., 2022 (13)		Case report	WES	1 family, 3 patients	*FCN3*
4	Akbari et al., 2021 (14)		Case - control	WES	1 family, 2 patients	*MSH4*
5	Alvi et al., 2020 (15)		Case - control	TGS and WES	1 family, 8 patients	*ALOX12B*
6	Safari et al., 2014 (16)		Case report	TGS	1 family, 1 patient	*NR5A1*
7	Rafaa et al., 2020 (17)	Iraq	Case - control	TGS	45 patients, 45 controls	*AMH*
8	Le Quesne et al., 2016 (18)	Lebanon	Case - control	WES	1 family, 2 patients	*STAG3*
9	Lacombe et al., 2006 (19)		Case - control	TGS	92 patients	*POF1B*
10	Smirin-Yosef et al., 2017 (20)	Palestine	Case - control	WES	1 family, 2 patients	*SPIDR*
11	de Vries et al., 2014 (21)		Case report	WES	1 family, 2 patients	*SYCE1*
12	Pierce et al., 2013 (22)		Case report	TGS	2 families, 2 patients	*LARS2*
13	Zangen et al., 2011 (23)		Case report	WES	1 family, 5 patients	*PSMC3IP*
14	Caburet et al., 2014 (24)		Case report	WES	1 family, 4 patients	*STAG3*
15	Al-Ajoury et al., 2015 (25)	Syria	Case - control	TGS	80 patients, 200 controls	*GDF9*
16	Al-Ajoury et al., 2015 (26)		Case - control	TGS	65 patients	*BMP15*
17	Bouali et al., 2017 (27)	Tunis	Case report	TGS	1 family, 5 patients	*MCM8*
18	Bouali et al., 2016 (28)		Case - control	TGS	125 patients, 200 controls	*NOBOX, BMP15, GDF9*
19	Lakhal et al., 2012 (29)		Case - control	TGS	55 patients, 100 control	*SF1, WNT4*
20	Lakhal et al., 2009 (30)		Case report	TGS	1 patient	*BMP15*
21	Ojeda et al., 2011 (31)		Case - control	TGS	87 patients	*CKDN1B*
22	Oral et al., 2019 (32)	Turkey	Case - control	TGS	86 patients, 26 control	*FSHR, NR5A1, PDPK1, POF1B*
23	Bramble et al., 2016 (33)		Case report	WES	1 family, 2 patients	*FSHR*
24	Haddar et al., 2022 (34)	Turkey and North African	Case - control	WES	375 patients	*C18orf53, HELQ, SWI5, SAPATA33, NLRP11, CCDC150, CCDC185*
25	Al-Agha et al., 2018 (35)	Yemen	Case - control	WES	1 family, 4 patients	*PSMC3IP*

TGS, Targeted gene panel sequencing; WES, whole exome sequencing.

### 3.2 Quality of the eligible studies

**Figure 2:**
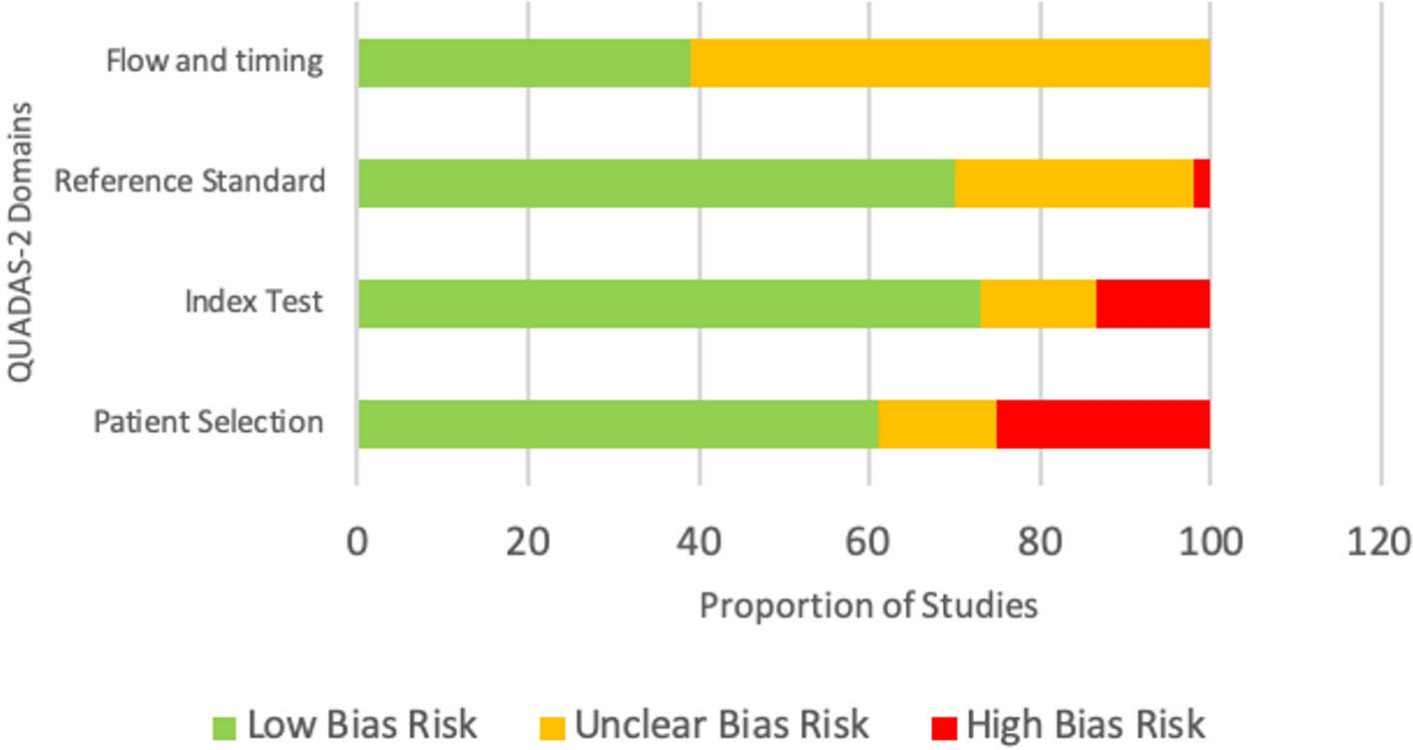
Risk of bias assessment using the QUADAS-2 tool.

The risk of bias assessment in the four domains of the QUADAS-2 tool is shown in **Figure 2**. The patient selection domain had the highest risk of bias, with 25% of studies showing a high risk of bias. This was expected, as most of the included articles were case reports with preselected participants. The remaining three domains had mainly a low risk of bias.

### 3.3 Reported genetic variants

In the 25 included articles, there were 1,080 non-syndromic POI participants from 10 familial/case report studies and 15 case-control studies. In total, seventy-nine POI-associated variants were reported in 25 genes. Consanguinity was observed in all the family studies. Genetic testing was through direct gene sequencing (n= 6), targeted gene panel sequencing (n=7), whole genome sequencing (N=2), whole exome sequencing (n=6), or homozygosity mapping and exome sequencing (n= 2). In most cases, the genotype was confirmed by Sanger sequencing.

The 79 POI-causing variants were reported from ten MENA countries: Tunisia, Yemen, Iran, Turkey, Palestine, Lebanon, Syria, Morocco, Iraq, and in Arab Israelis. No POI cases were reported from Algeria, Jordan, Bahrain, Qatar, Mauritania, Libya, Sudan, Somalia, Egypt, Oman, the Comoros Islands, Emirates, Djibouti, Afghanistan, and Cyprus. **Table 2** lists the genes reported in the included articles.

Of the 79 variants, 46 variants were rare, while 33 were common polymorphisms. Six polymorphisms were shared in *GDF9* (c.447C>T, c.546G>T) in Tunisian and Syrian patients. In *BMP15*, c.-9C>G and c. 308 A>G were shared among Tunisian and Iranian patients and c.852C>T was shared among Tunisian and Syrian patients. Finally, c.538G>A was shared among all three cohorts.

**Figure 3:**
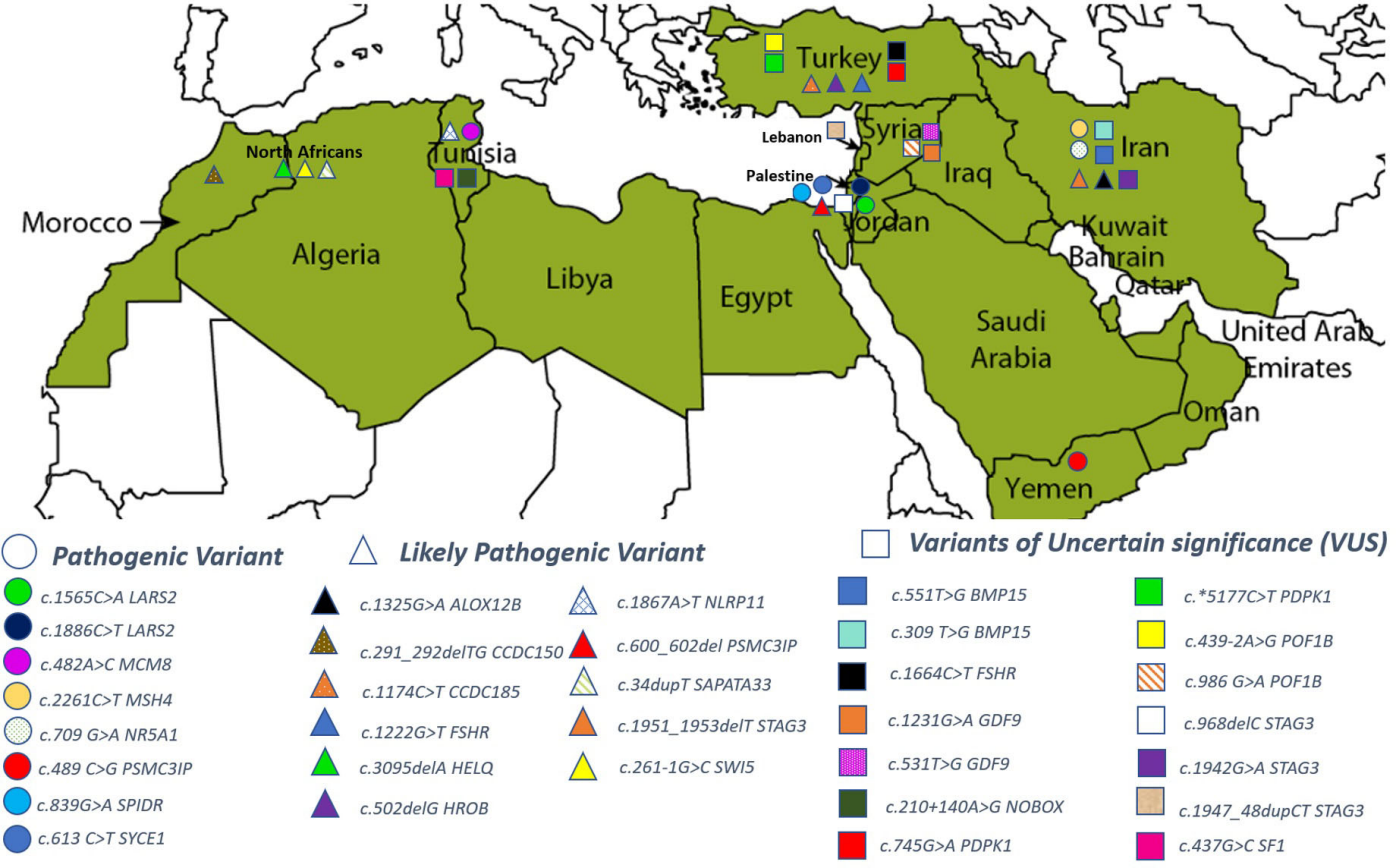
Geographical distribution of POI-associated genetic variants in the MENA region.

Of the 46 rare variants, 19 were reported pathogenic or likely pathogenic according to ACMG/AMP guidelines and the ClinVar database (**Table 3**). In the ClinVar database, seven variants were reported as pathogenic, nine as likely pathogenic, one as a variant of uncertain significance (VUS), and two had no data. However, following the ACMG/AMP guidelines, only two of the 19 variants were found as pathogenic, three as likely pathogenic, seven as VUS, and seven had no data. Most of these variants were detected in the patients in homozygous state (79%, 15 variants), while four variants (21%) were seen in the heterozygous state. **Table 4** shows variants reported as VUS by either ACMG/AMP or ClinVar or both. **Figure 3** shows the geographical distribution of POI-associated variants in the MENA region.

**Table 3:** Pathogenic and likely pathogenic variants reported in POI patients in MENA populations according to ACMG/AMP criteria and ClinVar.

Gene	Genomic Location	Variant	Amino acid change	Zygosity	gnomAD MAF	ACMG/AMP classification	ClinVar	Country	Sample description	Ref.
*STAG3*	7q22.1	c.1951_1953del	p. Leu652del	Homozygous	NA	NA	LP	Iran	1 Family (3 POI)	(12)
*MSH4*	1p31.1	c.2261C>T	p. Ser754Leu	Homozygous	0.000036	VUS (PM1, PP3)	**P**	Iran	1 family (2 POI)	(14)
*ALOX12B*	17p13.1	c.1325G>A	p. Arg442Gln	Homozygous/Heterozygous	0.00001	LP	VUS	Iran	1 family (8 POI)	(15)
*NR5A1*	9q33.3	*c.709 G>A*	p. Gly91ser	Heterozygous	NA	VUS	**P**	Iran	1 family (1 POI)	(16)
*SPIDR*	8q11.21	c.839G>A	p. W280*	Homozygous	NA	VUS	**P**	Palestine	1 family (2 POI)	(20)
*SYCE1*	10q26.3	c.613C>T	p. Gln205Ter	Homozygous	NA	**P**	**P**	Palestine	1 Family (2 POI)	(21)
*LARS2*	3p21.31	*c.1886C>T*	p. Thr629Met	Homozygous	0.00006014	LP	**P**	Palestine	2 Families (2 POI)	(22)
*LARS2*	3p21.31	c.1565C>A	p. Thr522Asn	Heterozygous	0.001	VUS (9PM1, PM2	**P**	Palestine	2 Families (2 POI)	
*PSMC3IP*	17q21.2	c.600_602del	p. Glu201del	Homozygous	NA	NA	LP	Palestine	1 family (5 POI)	(23)
*MCM8*	20p12.3	c. 482A>C	p. His161Pro	Homozygous/Heterozygous	NA	VUS (PM1, PM2, BP1)	**P**	Tunisia	1 family (5 POI)	(27)
*FSHR*	2p16.3	c.1222G>T	p. Asp408Tyr	Homozygous	NA	LP	NA	Turkey	1 family (2 POI)	(33)
*CCDC150*	2q33.1	c.291_292delTG	p. Cys97Ter	Homozygous	NA	NA	LP	Morocco	375 patients	(34)
*CCDC185*	1q41	c.1174C>T	p. Gln392Ter	Homozygous	NA	VUS (PM2, PP3)	LP	Turkey		
*HROB*	17q21.31	c.502delG	p. Glu168ArgfsTer35	Homozygous	NA	NA	LP	Turkey		
*HELQ*	4q21.23	c.3095delA	p. Tyr1032SerfsTer4	Homozygous	NA	NA	LP	North African		
*NLRP11*	19q13.42-q13.43	c.1867A>T	p. Arg623Ter	Heterozygous	NA	VUS (PM2)	LP	Tunisia		
*SPATA33*	16q24.3	c.34dupT	p. Cys12LeufsTer2	Homozygous	NA	NA	LP	North African		
*SWI5*	9q34.11	c.261-1G>C	–	Homozygous	NA	NA	LP	North African		
*PSMC3IP*	17q21.2	c.489 C>G	p. Tyr163Ter	Homozygous	NA	**P**	NA	Yemen	1 family (4 POI)	(35)

P, pathogenic; LP, likely pathogenic; VUS, variant of uncertain significance; NA, not applicable; Ref, references; ACMG, American College of Medical Genetics and Genomics; AMP, Association for Molecular Pathology; gnomAD, Genome Aggregation Database; MAF, Minor Allele Frequency; MENA, Middle East and North Africa; POI, primary ovarian insufficiency.

**Table 4:** Rare variants of uncertain significance reported in POI patients in MENA populations.

Gene	Genomic Location	Variant	Amino acid change	Molecular consequence	Zygosity	Country	gnomAD MAF	Clin-Var	ACMG/AMP classification	Ref.
*BMP15*	Xp11.22	c.309T>G	p.N103K	Missense	Heterozygous	Iran	NA	NA	VUS (PM2, BP4)	(11)
		c.551T>C	p.M184T							
*STAG3*	7q22.1	c.1942G>A	p.A648T	Missense	Homozygous	Iran	NA	VUS	VUS (PM1, PM2, BP1)	(12)
		c.1947_48dupCT	p.Y650Sfs*22	Frameshift	Homozygous	Lebanon	NA	NA	NA	(18)
		c.968delC	F187fs*7	Frameshift	Homozygous	Palestine	NA	NA	NA	(24)
*POF1B*	Xq21.1	c.986G>A	p.R329Q	Missense	Heterozygous	Syria	0.0021	NA	VUS (PM1,BS1)	(19)
*GDF9*	5q31.1	c.1231G>A	p. D411N	Missense	Heterozygous	Syria	NA	NA	VUS (PM1, PM2, BP1)	(25)
		c.531T>G	p. N177K	Missense	Heterozygous	Syria	NA	NA	VUS (PM1, PM2, BP1)	
*NOBOX*	7q35	c.210 + 140A>G	--	Intronic	Homozygous/heterozygous	Tunisia	0.00016	NA	NA	(28)
*SF1*	11q13.1	c.437G>C	G146A	Missense	NA	Tunisia	NA	NA	VUS (PM1, PM2, PP3)	(29)
*PDPK1*	16p13.3	c.745G>A	p.V249I	Missense	Heterozygous	Turkey	0.0000338	NA	VUS (PM1)	(32)
		c.*5177C>T	--	3’UTR	Heterozygous	Turkey	NA	NA	NA	
*POF1B*	Xq21.1	c.439-2A>G	--	Intronic	Heterozygous	Turkey	NA	NA	NA	(32)
*FSHR*	2p16.3	c.1664C>T	p.T555I	Missense	Heterozygous	Turkey	0.00004	VUS	VUS (PM1, PM2, PP3)	(32)

VUS, variant of uncertain significance; gnomAD, Genome Aggregation Database; MAF, Minor Allele Frequency; ACMG, American College of Medical Genetics and Genomics; AMP, Association for Molecular Pathology; PM1, Located in a mutational hot spot and/or critical and well-established functional domain (e.g.; active site of an enzyme) without benign variation; PM2, Absent from controls (or at extremely low frequency if recessive) in Exome Sequencing Project; 1000 Genomes Project; or Exome Aggregation Consortium; PP3, Multiple lines of computational evidence support a deleterious effect on the gene or gene product (conservation; evolutionary; splicing impact; etc.); BS1, Allele frequency is greater than expected for disorder; BP1, Missense variant in a gene for which primarily truncating variants are known to cause disease; BP4, Multiple lines of computational evidence suggest no impact on gene or gene product (conservation; evolutionary; splicing impact; etc.); NA, not available; Ref, references; MENA, Middle East and North Africa; POI, primary ovarian insufficiency.

### 3.4 Clinical phenotypes

**Table 5** details the phenotype-genotype correlations in the 1,080 patients from the 25 included articles. High FSH and LH levels were seen in all POI patients. All patients presented with normal karyotyping and negative *FMR1* premutation, so met eligibility criteria for unexplained non-syndromic POI. All patients presented with either primary or secondary amenorrhea. Where pelvic ultrasound data were available, most subjects had atrophic ovaries with diminished follicle reserve, small uteri, and bilateral streak gonads. Three studies report infertility in 3 males, all of whom were carriers of the pathogenic variants reported (*PSMC3IP*, *NR5A1*, and *STAG3* variants). No clear phenotype-genotype correlations were observed.

**Table 5:** Genotype-phenotype correlations reported for the pathogenic and likely pathogenic variants in the MENA region.

Gene	Menses	Hormonal		Ultrasound			Comments	Ref.
		↑FSH	↑LH	Atrophic ovaries	Atrophic uterus	Reduced follicle reserve		
*STAG3*	PA	100% (7/7 subjects)	100% (7/7 subjects)	100% (7/7 subjects)	–	–	A male carrier diagnosed with non-obstructive azoospermia	(12)
*MSH4*	SA	100% (2/2 subjects)	100% (2/2 subjects)	100% (2/2 subjects)	–	–	–	(14)
*ALOX12B*	SA	22% (2/9 subjects)	22% (2/9 subjects)	–	–	–	88% (6/9 subjects) were on estrogen replacement. Male carrier diagnosed with ichthyosis 1/1	(15)
*NR5A1*	SA	100% (2/2 subjects)	100% (2/2 subjects)	–	–	–	A male carrier diagnosed with non-obstructive azoospermia 1/1	(16)
*SPIDR*	PA	100% (2/2 subjects)	100% (2/2 subjects)	–	100% (2/2 subjects)	–		(20)
*SYCE1*	PA	100% (2/2 subjects)	50% (1/2 subjects)	100% (2/2 subjects)	100% (2/2 subjects)	100% (2/2 subjects)	–	(21)
*LARS2*	–	100% (2/2 subjects)	50% (1/2 subjects)	100% (2/2 subjects)	100% (2/2 subjects)	–	–	(22)
*PSMC3IP*	PA	100% (9/9 subjects)	100% (9/9 subjects)	100% (9/9 subjects)	100% (9/9 subjects)	–	A male carrier diagnosed with non-obstructive azoospermia 1/1	(23, 35)
*MCM8*	PA	100% (5/5 subjects)	100% (5/5 subjects)	80% (4/5 subjects)	80% (4/5 subjects)	–	–	(27)
*FSHR*	2 PA/1 SA	100% (3/3 subjects)	100% (3/3 subjects)	100% (2/2 subjects)	100% (2/2 subjects)	100% (2/2 subjects)	–	(33)

PA, primary amenorrhea; SA, secondary amenorrhea; FSH, follicle-stimulating hormone; LH, luteinizing hormone; ↑, elevated; Ref, references.

## 4 Discussion

Over 80 gene variants have been reported to be causative for POI worldwide (36). In this systematic review conducted on the MENA populations, we captured 79 genetic variants in 25 genes from 25 eligible studies comprising 1,080 cases. Forty-six variants were rare, while 33 were common polymorphisms. Among the 46 rare variants, 19 were pathogenic or likely pathogenic according to ACMG/AMP guidelines and the ClinVar database. Among the 25 reported genes, *BMP15* and *STAG3* were most frequently reported in MENA populations, with *BMP15* reported in four articles and *STAG3* in three articles. The most clinically relevant findings included the pathogenic variants in *SYCE1*, *LARS2*, *MCM8*, and *NR5A1* and the first report of pathogenic *PSMC3IP* variants in association with POI in two Arab cases (24, 26). The segregation of pathogenic loss of function variants in *PSMC3IP*, *NR5A1*, and *STAG3* with POI in 13 female carriers and non-obstructive azoospermia (NOA) in three male carriers in the studied pedigrees suggested a significant role of these variants in infertility in both genders (13, 17, 24, 36).

*PSMC3IP* encodes PSMC3-interacting protein or HOP2, whose loss of function is associated with ovarian dysgenesis and insufficiency. PSMC3IP plays a role in meiosis and DNA repair (37). In animal models, *PSMC3IP* knockout results in extreme loss of gametogenesis (38). The pathogenic variant in *PSMC3IP* (c.600_602del, p.Glu201del) in POI was first reported in 2011 in five females in a Palestinian family, all of whom were homozygous carriers with primary amenorrhea and undetectable ovaries (23). This remained the only report until 2018, when a homozygous stop-gain variant (c.489C>G,p.Tyr163Ter) was reported in four affected Yemini sisters and their brother (35), the sisters presenting with primary amenorrhea, delayed puberty, and very low AMH levels and their affected brother presenting with NOA. This prompted a thorough evaluation of *PSMC3IP* function. In 2019, compound heterozygous variants (c.496_497delCT, p.Arg166Alafs; c.430_431insGA, p.Leu144Ter) were detected in a woman in a French cohort of 33 POI patients (39). A Fujian woman with POI presented with the same phenotype of primary amenorrhea and similarly harbored a compound heterozygous variant (c.597 + 1G>T and c.268G>C) (40). A woman of Italian origin with secondary amenorrhea was also recently reported to possess biallelic variants (c.206_208delAGA and c.189 G>T) in *PSMC3IP*, thus expanding the phenotypic spectrum of *PSMC3IP* in association with POI. Interestingly, the sister of the affected female in this latter study also carried both variants but presented with normal menses and hormonal levels except for low AMH and infertility (41).

*STAG3* encodes a subunit of cohesin, a protein complex that functions in chromosome segregation during meiosis (24). *STAG3* variants are well established in POI, with reports in over 16 different patients from unrelated families (37). Interestingly, a recent study of familial infertility identified a homozygous mutation in a POI female and her NOA brother (42). Similarly, a study of an Iranian family of one POI female and her two NOA brothers identified double homozygous mutations in *STAG3*, one of which was predicted to be likely pathogenic according to ClinVar (c.1951_1953del, p. Leu652del) and the other a VUS according to ACMG/AMP guidelines (c.1942G>A, p.Ala648Thr) (12). We additionally found two VUSs in *STAG3* (c.1947_48dupCT, p. Y650Sfs*22; c.968delC, p. F187fs*7) in Lebanese and Palestinian patients (18, 24). Additionally, a likely homozygous pathogenic variant (c.1222G>T, p. Asp408Tyr) and a heterozygous VUS (c.1664C > T, p.Thr555Ile) in *FSHR* were identified in Turkish probands (32, 33). *FSHR* mutations are implicated with ovarian dysgenesis and POI in an autosomal recessive manner, with over 19 published research articles and case reports (37).

The pathogenic variants reported in ClinVar are *SYCE1*, *LARS2*, *MCM8*, and *NR5A1*. *SYCE1* encodes synaptonemal complex central element protein 1 (SYCP1), an element of the synaptonemal complex, which binds to a homolog associated in meiosis (synapsis) before crossover (43). Each homolog connected through SCYP1 contains SYCE1 and SYCE2, which are crucial for synaptonemal complex assembly and completion of oogenesis (44). Several studies have showed that female infertility can be caused by a failure in building a synaptonemal complex, a potential causative of primary ovarian insufficiency (45). The *SYCE1* c.613C>T nucleotide change was the first to be reported to cause POI in two daughters from a Palestinian family in 2014 (21). A year later, the c.197-2 A > G variant was reported in association with azoospermia in two first cousins originally from Iran (46).

A mutation in *LARS2*, which encodes mitochondrial leucyl-tRNA synthetase, is also potentially causative for POI. *LARS2* is clearly associated with molecular defects in mitochondrial function that contribute to the development of POI (47). The *LARS2* variant p.Thr522Asn has been reported in three individuals from Palestine with severe hearing loss and POI associated with Perrault syndrome (22, 48). Interestingly, *in vitro* functional studies of the p. Thr522Asn variant show that the mutation causes a significant decrease in protein activity, which is probably responsible for its deleterious impact (22, 49). Moreover, minichromosomal maintenance protein-8 (MCM8) is known to be an initiator of eukaryotic genome replication. Indeed, MCM8 plays a vital role in meiosis and DNA double-stranded break repair, and it contributes to menopause (50). In 2017, the homozygous c.482A>C mutation in *MCM8* was first reported to be deleterious, segregating in a Tunisian family with several affected siblings with POI. *In vitro* analyses in the same study showed *that MCM8* knockout results in small gonads and disrupted follicle development, so may be related to the primary ovarian insufficiency phenotype (27). Another study showed that *MCM8* frameshift mutations are pathogenic and may directly cause POI (51). Finally, *NR5A1* is associated with a wide spectrum of phenotypes including sex determination, XY sex gonadal dysgenesis, adrenocortical insufficiency, and ovarian failure (52). p.Gly91Ser was the first *NR5A1* variant to be reported in two siblings, a man with azoospermia and his sister with POI (16). NR5A1 is likely to play a pivotal role in regulating ovary and testis genes, but there has yet to be a functional study to confirm its role (53).

Beyond the exploration of causative mutations linked to Primary Ovarian Insufficiency (POI), numerous studies have delved into the investigation of common polymorphisms within Middle Eastern and North African (MENA) populations. Among these, some of the most frequently reported common polymorphisms are found in genes such as NOBOX, with 15 distinct variants (PMID 26848058), BMP15 with 11 variants(PMID 3523244), GDF9 featuring 4 variants(PMID 16278619), AMH contributing 2 variants(PMID 33344660), CKDN1B with 1 variant(PMID21575944), and FCN3 presenting a single variant(PMID 34641644). To provide a more comprehensive perspective, the genes NOBOX, BMP15, GDF9, AMH, CKDN1B, and FCN3 each play crucial roles in ovarian function and reproductive health. NOBOX is a key player in early folliculogenesis, orchestrating the development of ovarian follicles. BMP15 and GDF9 are essential for promoting the growth and maturation of ovarian follicles, vital to produce mature eggs. AMH, on the other hand, helps assess ovarian reserve and regulates the development of ovarian follicles. CKDN1B, though less directly tied to ovarian function, influences cell cycle regulation and may have subtle effects on ovarian health. In contrast, FCN3, while not a direct participant in ovarian function, is related to the immune system, and variations in immune-related genes can indirectly affect reproductive health. Genetic variants in these genes can lead to diverse effects on ovarian function, potentially contributing to conditions such as Primary Ovarian Insufficiency (POI) and impacting fertility in Middle Eastern and North African populations (PMID 24782009).

Four of the common *BMP15* variants observed in MENA populations (c.-9C>G, c.308A>G, c.538G>A, and c.852C>T) have also been reported in Indian and Brazilian patients. Three of these (c.-9C>G, c.308A>G, and c.852C>T) were significantly associated with ovarian failure (54, 55): the c.-9C>G variant was associated with anovulation or infertility in a Spanish cohort of polycystic ovarian syndrome (PCOS) patients (55) but not in Chinese patients (56). One of the *GDF9* variants found in POI patients from MENA have also been reported in different cohorts with different ancestries, namely *GDF9* c.447C>T, which was a risk factor for POI in an Indian cohort (54) but not in Chinese Hui or Mexican patients (56, 57). The presence of common POI polymorphisms in the same genes that contain rare disease-causing mutations is an interesting finding, as it indicates a hereditary predisposition and may help in defining the genetic role of these genes in developing the disease.

POI demands early diagnosis to prevent irreversible damage to a patient’s fertility. According to recent studies, timely identification of POI is crucial, as it enables healthcare professionals to implement appropriate interventions, thereby preserving reproductive capabilities (58–60). Genetic testing assumes a pivotal role in this process, as it helps identify any underlying genetic abnormalities that contribute to POI. By understanding the genetic variants of the condition, healthcare providers can offer tailored treatment options and inform patients about their reproductive prognosis and potential risks. Hormone replacement therapy (HRT) is one possible intervention to manage hormonal imbalances and alleviate associated symptoms in POI, thereby potentially restoring fertility. Additionally, fertility preservation techniques, such as oocyte or embryo cryopreservation, provide an opportunity for future conception. Early diagnosis coupled with genetic testing and the availability of suitable interventions like HRT and fertility preservation are extremely important for mitigating the impact of POI and ensuring that patients receive appropriate support for their fertility and overall well-being (61).

## 5 Conclusions

Technological advances have facilitated the discovery of several new genes that may cause POI, partially explaining its heterogeneity. Several case studies have identified potential candidate genes that might cause POI, and, in many cases, this was supported by familial segregation analysis. Further functional studies are required to assess the underlying molecular mechanisms and pathways responsible for POI. Knowledge of the genetic basis of POI in the MENA region is expected to facilitate early diagnosis of POI patients and thus the early implementation of therapeutic interventions, paving the way for precision medicine options in specific patient populations.

## Data availability statement

The original contributions presented in the study are included in the article/supplementary material. Further inquiries can be directed to the corresponding author.

## Author contributions

AA: Data curation, Investigation, Methodology, Writing – original draft. TA-B: Data curation, Investigation, Methodology, Writing – original draft. MA-S: Conceptualization, Resources, Supervision, Validation, Visualization, Writing – review & editing. AMA: Conceptualization, Formal analysis, Funding acquisition, Project administration, Supervision, Writing – review & editing.
